# The Role of Electrode-Site Placement in the Long-Term Stability of Intracortical Microstimulation

**DOI:** 10.3389/fnins.2021.712578

**Published:** 2021-09-08

**Authors:** Ramya L. Saldanha, Morgan E. Urdaneta, Kevin J. Otto

**Affiliations:** ^1^J. Crayton Pruitt Family Department of Biomedical Engineering, University of Florida, Gainesville, FL, United States; ^2^Department of Neuroscience, University of Florida, Gainesville, FL, United States; ^3^Department of Materials Science and Engineering, University of Florida, Gainesville, FL, United States; ^4^Department of Neurology, University of Florida, Gainesville, FL, United States; ^5^Department of Electrical and Computer Engineering, University of Florida, Gainesville, FL, United States

**Keywords:** brain-machine interfaces, neuroprosthetics, intracortical microelectrodes, microstimulation, electrode design

## Abstract

Intracortical microelectrodes are neuroprosthetic devices used in brain-machine interfaces to both record and stimulate neural activity in the brain. These technologies have been improved by advances in microfabrication, which have led to the creation of subcellular and high-density microelectrodes. The greater number of independent stimulation channels in these devices allows for improved neuromodulation selectivity, compared to single-site microelectrodes. Elements of electrode design such as electrode-site placement can influence the long-term performance of neuroprostheses. Previous studies have shown that electrode-sites placed on the edge of a planar microelectrode have greater chronic recording functionality than sites placed in the center. However, the effect of electrode-site placement on long-term intracortical microstimulation (ICMS) is still unknown. Here, we show that, in rats chronically implanted with custom-made planar silicon microelectrodes, electrode-sites on the tip of the device outperformed those on both the edge and center in terms of the effect per charge delivered, though there is still a slight advantage to using edge sites over center sites for ICMS. Longitudinal analysis of ICMS detection thresholds over a 16-week period revealed that while all sites followed a similar trend over time, the tip and edge sites consistently elicited the behavioral response with less charge compared to center sites. Furthermore, we quantified channel activity over time and found that edge sites remained more active than center sites over time, though the rate of decay of active sites for center and edge sites was comparable. Our results demonstrate that electrode-site placement plays an important role in the long-term stability of intracortical microstimulation and could be a potential factor to consider in the design of future intracortical electrodes.

## Introduction

Bidirectional brain-machine interfaces (BMIs) have the potential to restore function in patients with neurological disabilities by recording and stimulating selected neurons (Lebedev and Nicolelis, 2006). While recordings alone could be used to control a robotic arm, for example, a closed-loop system that involves stimulating sensory feedback as well could enable more intricate movement (Bensmaia and Miller, 2014). Intracortical microstimulation (ICMS), while invasive, can achieve greater specificity than other methods of stimulation as a result of advances in microfabrication techniques that have allowed for the development of high-density microelectrodes with smaller electrode-sites (Seymour et al., 2017). However, intracortical microelectrodes often have poor chronic performance as a result of the foreign body response (FBR), which leads to the formation of fibrous tissue around the device and neuronal cell death over time (Biran et al., 2005; Kozai et al., 2015). Developing a system that performs well over a long period of time is imperative to improving this technology and translating it to clinical use.

Much work has been done to investigate methods to mitigate the FBR to improve performance by changing the device shape and size (Karumbaiah et al., 2013; Luan et al., 2017), site geometry (Park et al., 2018), and device coating (He et al., 2006). These studies have revealed that changing elements of electrode design can affect the long-term tissue response, and therefore the recording and stimulation efficacy of implantable devices. This strategy has been successful in improving stimulation performance in areas such as deep brain stimulation, in which changes in electrode geometry have enabled greater selectivity and efficiency while avoiding off-target effects (Butson and McIntyre, 2005; Howell et al., 2015). The design of high-density planar microelectrode arrays similarly provides an opportunity to optimize chronic stability by changing the site size, depth, and placement of electrode-sites on a shank. Previous work from our group (Lee et al., 2017) found that electrode-site placement can influence the long-term function of intracortical electrodes. Specifically, sites placed on the edge outperformed those in the center in nearly every case in terms of intracortical recordings, with a higher SNR and number of active sites on average. The effects of electrode-site placement on microstimulation, however, have not been studied.

Here, we investigated the role of electrode-site placement by implanting a single-shank silicon microelectrode array with sites on the edge, center, and tip. ICMS detection thresholds and voltage transients were measured for 16 weeks post implantation (WPI). This data was used to determine chronic microstimulation performance and number of active sites for edge, center, and tip sites over time. We found that edge sites generally performed more efficiently than corresponding center sites at the same depth, eliciting the desired response with less charge especially in the long term. Furthermore, we observed an upward trend in performance with increasing cortical depth. These results suggest that electrode-site placement is a factor of electrode design that can be targeted to improve longitudinal microstimulation performance.

## Results

**FIGURE 1:**
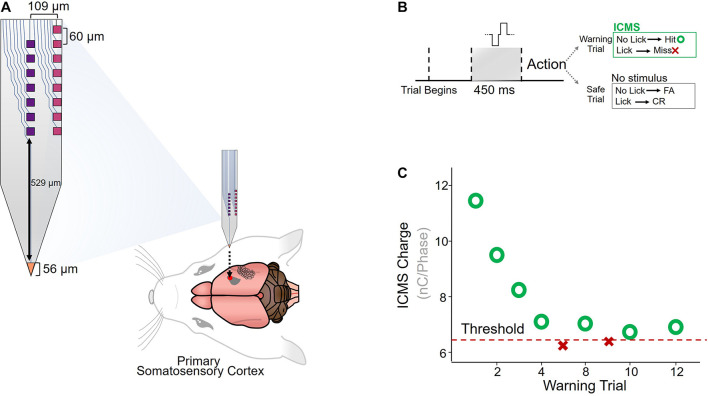
Implantation and behavioral paradigm. **(A)** Diagram of custom-made planar silicon microelectrode array (Lee et al., 2017), implanted in the primary somatosensory cortex of Sprague-Dawley rats (*N* = 6). **(B)** A conditioned-avoidance behavioral paradigm was used to measure ICMS detection thresholds. Rats were trained to briefly stop drinking after an ICMS stimulus was detected, and failure to stop drinking resulted in a small shock applied through the drinking spout. **(C)** Adaptation protocol for determining detection thresholds.

Custom-made planar silicon microelectrode arrays were chronically implanted in the primary somatosensory cortex of six adult Sprague-Dawley rats (Figure 1A). The microelectrode array had seven electrode-sites down the center, eight on the edge, and one on the tip of the device (Lee et al., 2017). ICMS detection thresholds were measured using a conditioned-avoidance behavioral paradigm, in which water-deprived rats were trained to stop drinking briefly after detecting the ICMS stimulus (Figure 1B). A mild percutaneous shock was applied through the drinking spout if the rat failed to correctly detect the stimulus. Each ICMS stimulus amplitude was modulated according to the animal’s response to the previous stimulus (decreased after a hit and increased after a miss) until a detection threshold was determined (see section “Materials and Methods”; Figure 1C).

### ICMS Detection Threshold Charge Varies With Electrode-Site Placement

**FIGURE 2:**
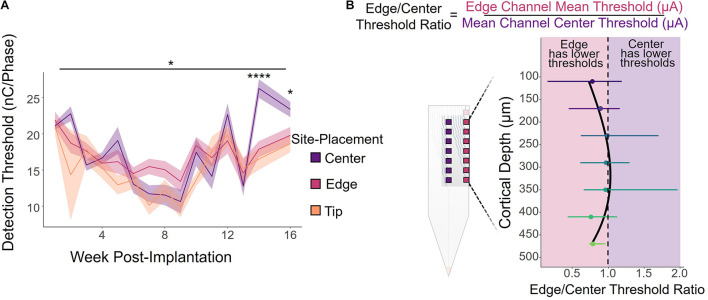
ICMS detection thresholds by electrode-site placement. **(A)** Average detection thresholds for center, edge, and tip sites over a 16-week period for all six animals. Shading indicates standard error. *p ≤ 0.05, ****p ≤ 0.0001 **(B)** Edge/Center threshold charge ratio by cortical depth, calculated by dividing the average threshold of an edge channel by the threshold of the corresponding center channel at the same cortical depth, weekly per animal. Channels toward the middle of the array (cortical depths of 240–360 μm) had ratios near 1, indicating that there was little or no difference in the charge required to elicit a response between the center and edge sites. However, the deeper and superficial channels had ratios less than 1, indicating that edge sites had lower thresholds on average. Error bars indicate standard deviation.

The primary objective of this study was to determine what, if any, effect electrode-site placement has on ICMS performance. We first analyzed how electrode-site placement affects long-term ICMS stability. Figure 2A shows the longitudinal threshold data for center, edge, and tip sites over the entire 16-week period, averaged across all six animals. The charge necessary to elicit a detection threshold for all sites followed a similar overall trend. During the first 9 weeks post-implantation, the average threshold across all channels decreased by 55.5%, then increased by 173% over the remaining 7 weeks. We also found that site placement had a statistically significant effect on thresholds after using time as a covariate [Figure 2A; *F*(2,711) = 3.19, *p* = 0.0416, ANCOVA]. While the tip site consistently had the lowest thresholds of the three groups, edge site thresholds seemed to remain the most stable throughout the entire period. The difference in thresholds between the three groups at 14 and 16 weeks post-implantation was statistically significant (week 14: *F*(2,29) = 17.0, *p* = 1.32 × 10^–5^, one-way ANOVA; week 16: *F*(2,27) = 3.76, *p* = 0.0361, one-way ANOVA), with center channels requiring more charge to elicit the behavioral response than both edge and tip channels.

It is important to note, however, that cortical depth plays a role in ICMS performance, as shown in previous studies (Bak et al., 1990; Tehovnik and Slocum, 2009; Koivuniemi et al., 2011; Aberra et al., 2018; Urdaneta et al., 2021). Therefore, we compared the performance of edge and center channels at the same cortical depth by calculating the ratio between the thresholds for an edge site and the corresponding center site at each cortical depth, computed weekly for each animal. Figure 2B shows the average edge to center threshold ratio over the 16-week period for all animals, where a ratio less than 1 indicates that edge channels had lower thresholds and were therefore more efficient than center channels, and similarly a ratio greater than 1 represents performance with greater efficiency in center channels than edge channels. Error bars indicate standard deviation. While channels located at cortical depths of 240–360 μm had average ratios near 1, indicating no difference in performance between edge and center channels, edge channels were more effective at both deeper and superficial sites. At nearly all cortical depths, the edge to center ratio was less than 1, with an average ratio of 0.91 ± 0.34 across all channels.

### The Number of Active Sites Over Time Is Influenced by Site Placement

**FIGURE 3:**
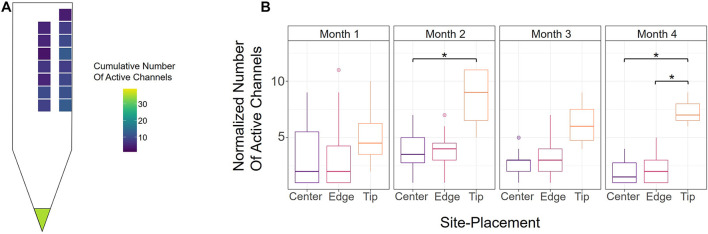
Active channels over time. **(A)** Heatmap of the average cumulative number of active channels for each site over the 16-week period across all six animals. An electrode-site was considered active if it could elicit a behavioral response below 30 nC/Phase during an experimental session. **(B)** Number of active center, edge, and tip channels for each month across all six animals, normalized by number of sites. *p ≤ 0.05

To further evaluate how electrode-site placement affects microstimulation performance, we assessed the number of active channels, defined as the site’s ability to elicit a response with charge below 30 nC/Phase during an experimental session (see section “Materials and Methods”). Figure 3A shows a heatmap of the average cumulative number of active sites over the 16-week period, indicating the average total number of thresholds obtained from each channel. The tip had the most active sites on average, though this may be due to its cortical depth relative to the other sites, for which we also see an increase in active sites as they approach deeper cortical layers. Between edge and center, the edge sites outperformed center sites in the average number of active sites at nearly every cortical depth, indicating that edge sites could produce the desired response at safe charges more often than center sites.

To determine how electrode-site placement affects long-term performance, we also calculated the number of active channels for each month. In order to properly compare the tip, edge, and center sites, however, the numbers of active sites were normalized to account for the different numbers of electrode-sites in the array (one tip, seven center, eight edge). Figure 3B shows the normalized numbers of active sites for each month from all animals. Consistent with Figure 3A, the tip site has the greatest number of active sites in every month, with a significant difference observed in months 2 and 4 (month 2; center vs. tip: *p* = 0.043, unpaired *t*-test with Dunn-Bonferroni correction; month 4; center vs. tip: *p* = 0.019, edge vs. tip: *p* = 0.021, unpaired *t*-test with Dunn-Bonferroni correction). Additionally, the number of active channels for all sites decreased gradually after the second month, which further demonstrates the decline in performance seen in Figure 2A over time. The results in Figure 3B show that there were 7.56% more active edge sites in month 2, 10.9% more active edge sites in month 3, and 13.8% more edge sites in month 4. To further assess the rate of decay in center and edge channel activity, we compared the slopes from the point of max channel activity (week 6) until the end of the study. This analysis showed that center sites decreased at a rate of −0.318 active channels/week while edge sites decreased at −0.296 active channels/week (linear regression slope from week 6 to week 16). The difference between the slopes for the active sites over time for center and edge was not statistically significant (*p* = 0.747, *t*-test for comparison of linear regression slopes). These analyses suggest that unlike detection thresholds (Figure 2A), the rate of decay of active sites for center and edge sites was comparable and therefore not significantly influenced by electrode-site placement.

### Voltage Transients Are Not Significantly Affected by Electrode-Site Placement

**FIGURE 4:**
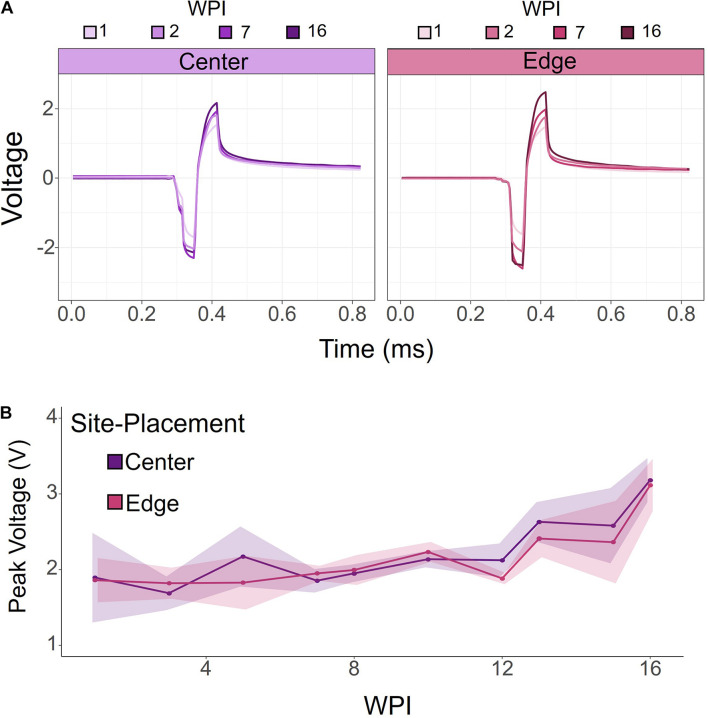
Voltage transients of contiguous center and edge channels over time. **(A)** Representative voltage transients of center and edge electrode-sites at the same cortical depth. Both center and edge channels show an increasing voltage transient over time. **(B)** Peak voltage for center and edge channels from all animals. Peak voltage increased over time for both center and edge channels. There was no significant difference between center and edge peak voltages. Shading indicates standard error.

To investigate how electrode-site placement affects long-term electrode stability for ICMS, voltage transients were measured to determine the maximum polarization at the electrode-electrolyte interface. Voltage transients were measured from each electrode in response to a symmetric biphasic pulse in awake rats prior to stimulation sessions. Figure 4A shows representative voltage transients from an edge and center site at the same cortical depth (410 μm) from one rat at selected points throughout the duration of the study. Overall, voltage transients increased over time, with the largest change occurring after 7 WPI. To further investigate this relationship, we measured voltage transients at least once per week for 16 weeks. Figure 4B shows the average peak voltages of contiguous center and edge electrode-sites for all animals over time. The peak voltages for center and edge sites follow a very similar trend, gradually increasing over time. There was no significance between the center and edge site peak voltages (for all tested weeks: *p* > 0.05, one-way ANOVA).

## Discussion

While microstimulation has the potential to treat many neurological disorders, a major limitation of this technology is the poor chronic performance of intracortical microelectrodes (Salatino et al., 2017). In this study, we show that electrode-site placement is an element of microelectrode design that can be modified to improve long-term microstimulation capabilities of implantable neuroprostheses. To determine the effects of electrode-site placement on microstimulation, we designed a custom-made microelectrode array with sites on the edge, center, and tip of a single shank. We found that sites on the edge of the device were more efficient than those in the center in terms of longitudinal ICMS detection thresholds and number of active channels, though the rate of decay of active sites and electrochemical characterizations of center and edge sites were very similar. We also observed that performance generally improves as cortical depth increases, a trend seen in similar studies (Bak et al., 1990; Tehovnik and Slocum, 2009; Koivuniemi et al., 2011; Aberra et al., 2018; Urdaneta et al., 2021).

Consistent with previous work investigating the role of electrode-site placement on recordings, we found that in most cases edge sites perform with greater efficacy than center sites for microstimulation, especially for long-term use. For instance, using the same microelectrode design, Lee et al., 2017 showed that edge sites are more efficient for intracortical recordings. Specifically, the fraction of active electrodes and SNR were almost always greater for edge sites than center sites, though both decrease with time. Our results show a similar trend between center and edge longitudinal ICMS detection thresholds and number of active sites over time.

The decrease in performance over time could be a result of the FBR, known to cause the formation of a glial encapsulation of the device, leading to a decay in recording capabilities (Biran et al., 2005; Kozai et al., 2015). Though the glial encapsulation may affect stimulation differently than it does recordings (Salatino et al., 2017), the greater decline in performance we noted in center sites could instead be caused by neuronal cell death that occurs over time as a result of implantation and chronic inflammation (Biran et al., 2005). Using finite element modeling, Skousen et al. (2011) found that TNF-a and MCP-1, pro-inflammatory cytokines, were more likely to be concentrated on the surface of the device rather than the edges, suggesting that there were greater amounts of these molecules present around the center sites of this single shank microelectrode array. Similarly, center shanks in Utah arrays have shown a more pronounced FBR and significantly lower SNR than edge shanks (Nolta et al., 2015; Joshi-Imre et al., 2019). These findings were attributed to the increased presence of inflammatory cytokines and nearby neuronal cell death as well. This phenomenon could be a potential explanation for our findings, where we saw a faster decline in performance in the center sites. Future studies are needed to determine how the tissue changes induced by these molecules affect microstimulation. Another possible explanation for the results observed here is the effective volume of tissue affected by center and edge sites. Center sites may be more limited in current spread than edge sites, and therefore reach smaller volumes of tissue. However, further studies are needed to investigate this phenomenon.

Despite differences in microstimulation capabilities observed between center and edge channels, electrochemical characterizations of the sites revealed nearly identical peak voltages from measurements of voltage transients over time. Similarly, Lee et al. (2017) found no difference in impedance measurements between center and edge sites. According to Ohm’s Law, voltage transients and impedance of the interface should covary since current-controlled stimulation was used here. These results together suggest that the decline in performance is not caused by electrochemical differences that could lead to electrode-site damage, but potentially by the surrounding tissue and neuronal changes. Because center sites had fewer active channels and required more charge to elicit the behavioral response over time than other sites, these results further indicate that the tissue response and nearby neuronal cell loss might have a larger effect on center sites than edge sites.

Interestingly, we also observed that deeper channels had lower detection thresholds than superficial channels, which is consistent with previous findings stating that deeper cortical layers are more sensitive to microstimulation (Bak et al., 1990; Tehovnik and Slocum, 2009; Koivuniemi et al., 2011; Aberra et al., 2018; Urdaneta et al., 2021). We observed a positive trend in performance toward deeper channels for both center and edge sites, and the tip had by far the greatest number of active sites and the lowest ICMS detection thresholds.

Although determining the effects of electrode-site placement on intracortical microstimulation is an important step in designing more effective neuroprostheses, more work must be done to address the limitations of the present study. Using a single electrode design across all animals, we found that edge sites carry a slight advantage over center sites in terms of long-term performance, so future studies could investigate how shanks of different widths affect center and edge site longitudinal microstimulation abilities. Decreasing the width of the shank would effectively move center sites closer to the edge, potentially reducing the differences we observed between the two. Lee et al. (2017) showed that for recordings, there were less pronounced differences in SNR between center and edge sites for narrow shanks, compared to the wide design used here as well. As noted previously, we found that cortical depth significantly affected electrode-site activity. The microelectrode array used here did not span all cortical depths, so future studies are needed to determine if these results hold for other cortical layers. Additionally, the FBR was not quantified in this study, so more work needs to be done to determine the extent to which the tissue damage affects microstimulation. The results presented here could have also been influenced by changes to the probe insulation material or electrode site materials during and after microstimulation and could be an important variable to consider (Prasad et al., 2014). While this is outside the scope of this project, these effects should be studied further.

The results presented here will lead to the design of intracortical microelectrodes that are more viable for chronic use, with implications for bidirectional brain-machine interfaces that are able to both record and stimulate critical neuron populations. Our results show that placing electrode-sites on the edge of microelectrode arrays could potentially prolong the active lifespan of these devices and provides further support that the shape and architecture of a device affects the foreign body response, which has been observed in several instances (Skousen et al., 2011; Nolta et al., 2015; Lee et al., 2017; Joshi-Imre et al., 2019). Furthermore, our findings on the effects of electrode-site placement along with channel depth has implications for the design of electrodes that take advantage of the specific anatomy of target areas of the brain. This approach, which has been used in the field of deep brain stimulation (Butson and McIntyre, 2005; Howell et al., 2015), will potentially lead to microelectrodes that are more effective in eliciting the desired response and using less charge, and could result in a device that is more suitable for long-term use. Additionally, the differences observed between different electrode-site placements may result in different perceptual qualities of microsimulation in future human studies (Hughes et al., 2020). As research in implantable neuroprostheses moves toward designing longer-lasting devices fit for human use, our findings will contribute to the design of intracortical microelectrodes that exhibit enhanced chronic performance and a more targeted approach for microstimulation.

## Materials and Methods

### Device Configuration

Six custom-made planar silicon microelectrode arrays (GP_1 × 16_249, NeuroNexus, Ann Arbor, MI, United States) with seven iridium oxide electrode-sites in the center, eight on the edge, and one on the tip of the device were used in this study and previously by Lee et al. (2014, 2017) (Figure 1A). Center and edge sites had a geometric surface area of 900 μm^2^ and the tip site had an area of 946 μm^2^. Each 16-channel device was approximately 2.2 mm long and 15 μm thick with a width of 249 μm. The electrodes had an insulation material of silicon dioxide. A flexible polyimide cable was used to connect the shank to a ZIF connector.

### Surgical Implantation

All surgeries and experiments were performed in accordance with the University of Florida’s Institutional Animal Care and Use Committee (IACUC). Surgeries were performed using aseptic techniques, and devices were sterilized with ethylene oxide and rinsed with sterile saline prior to implantation. Six adult male Sprague-Dawley rats (450–650 g, Charles River, Chicago, IL, United States) were anesthetized with 5% isoflurane (Zoetis, Parsippany, NJ, United States) in oxygen at 1.5–2 L/min. Meloxicam (1–2 mg/kg, SQ, Loxicom, Norbrook Laboratories, Newry, Northern Ireland) was administered subcutaneously and then the isoflurane was reduced to 1.5–3% for the duration of the procedure. A microdrill was used to prepare a 1 mm^2^ cranial window over the primary somatosensory cortex (0.5 mm, 3.5 mm) following a midline skin incision and periosteum removal. Four titanium bone screws (United Titanium, OH, United States) used for electrical grounding and headcap anchoring were placed by drilling burr holes. Following a durotomy, the microelectrode device was inserted 1600 μm at 100 mm/s using an automated micro-insertion system (PiLine M663, Physik Instrumente, Karlsruhe, Germany). Using a surgical microscope, the implantation depth was verified by confirming complete insertion of the most superficial electrode. Silicon elastomer (Kwik-Sil, WPI, Sarasota, FL, United States) was inserted into the craniotomy site, and headstage connectors were secured with layers of UV cured dental composite (DentalSource, CA, United States).

### Behavioral Paradigm

Intracortical microstimulation detection was evaluated using a conditioned avoidance behavioral paradigm (Heffner and Heffner, 1995; Koivuniemi et al., 2011). The animal’s activity was monitored using a custom RPvds code and a RZ5D Bioamp processor (Tucker Davis Technologies, Alachua, FL, United States). Water-deprived rats were presented with water through a drinking spout and trained to briefly stop drinking after an ICMS stimulus was applied. A mild percutaneous shock was delivered through the spout if the animal failed to stop drinking upon presentation of the stimulus. Each trial block consisted of one warning trial and four safe trials presented in a pseudo-random order. A trial was initiated only if the animal was in contact with the spout for more than 25% of a 200 ms window. The ICMS stimuli were only applied during warning trials, in which the animal had to avoid drinking for more than 20% of a 650 ms ICMS presentation phase to be considered a “hit” (Figure 1B). Safe trials were used to monitor licking behavior and discard trials in which the animal was not drinking for more than 20% of a 650 ms decision window (false alarm). If more than two false alarms were recorded within a block of five trials, the entire block was discarded and repeated. For each “hit,” the stimulus amplitude for the next warning trial was reduced. Similarly, the stimulus amplitude was increased each time the animal failed to stop drinking during a warning trial. As the number of warning trials increased, the change in stimulus amplitude decreased. A switch from “hit” to “miss” or vice versa was designated as a reversal. After three reversals, the ICMS detection threshold was calculated by averaging the previous five warning trials.

### Microstimulation Experiments

Experimental sessions began 5 days after surgery to allow for sufficient recovery and data was collected 5 times per week for 16 weeks post-implantation (WPI). During each session, ICMS detection thresholds were obtained from randomly selected channels until the animal was satiated. Microstimulation was administered using an IZ-32 stimulator with an LZ48-200 battery (Tucker-Davis Technologies, Alachua, FL, United States) to a single electrode-site on the device, with stimulus upper and lower bounds set manually. Cathode-leading charge-balanced symmetric waveforms with a phase duration of 0.2 ms, phase delay of 0.04 ms, and frequency of 320 Hz were used for all microstimulation experiments. The stimulus amplitude (μA) of detection thresholds were multiplied by the phase duration (ms) to report values in charge per phase (nC). The total charge delivered was limited to a maximum of 30 nC/phase (150 μA) for all experiments.

### Voltage Transient Measurements

Voltage transients were measured from each electrode-site prior to microstimulation at least once per week for 16 WPI. Symmetric biphasic pulses at 50 Hz with a 50 μs phase duration and amplitude of 5 μA were applied. Current-controlled stimulation was delivered using an IZ-32 stimulator with an LZ48-200 battery (Tucker-Davis Technologies, Alachua, FL, United States) to each electrode-site on the device. Data was acquired using the RZ5D Bioamp processor (Tucker Davis Technologies, Alachua, FL, United States).

### Statistics

For statistical reporting, the following significance levels were used: ^∗^*p* ≤ 0.05, ^∗∗^*p* ≤ 0.01, ^∗∗∗^*p* ≤ 0.001, ^****^p ≤ 0.0001. After verification of normality and homogeneity of variances, parametric tests were performed. In particular, analysis of variance in threshold charge between site placement groups was assessed using a one-way ANOVA. With time as a covariate, differences in threshold charge across the site placement groups were also analyzed with an ANCOVA. Pairwise comparisons for electrode-site activity were performed using a *t*-test with Bonferroni-Dunn correction. A linear regression was used to determine the slopes for the change in number of active sites over time for center and edge sites, and they were compared using a *t*-test. Statistical analyses were executed in R Statistical Software Version 4.0.1 (Vienna, Austria).

## Data Availability Statement

The raw data supporting the conclusions of this article will be made available by the authors, without undue reservation.

## Ethics Statement

The animal study was reviewed and approved by University of Florida Institutional Animal Care and Use Committee (IACUC).

## Author Contributions

KO sourced the funding. RS, MU, and KO conceived and designed the study and reviewed and edited the manuscript. MU performed the surgical implantations. RS and MU collected and analyzed the microstimulation data. RS wrote the manuscript. All authors contributed to the article and approved the submitted version.

## Conflict of Interest

The authors declare that the research was conducted in the absence of any commercial or financial relationships that could be construed as a potential conflict of interest.

## Publisher’s Note

All claims expressed in this article are solely those of the authors and do not necessarily represent those of their affiliated organizations, or those of the publisher, the editors and the reviewers. Any product that may be evaluated in this article, or claim that may be made by its manufacturer, is not guaranteed or endorsed by the publisher.
